# Cyclophosphamide for the Treatment of Refractory Immune Effector Cell-Associated Neurotoxicity Syndrome Following CD19-Targeted CAR T-Cell Therapy

**DOI:** 10.14740/jmc5211

**Published:** 2025-12-24

**Authors:** Austin Frisch, Loren Marino, Deena Alsaadi, Aditya Kasarabada, Gwen Hua, Germame Ajebo, Stephen Medlin, Zartash Gul

**Affiliations:** a Department of Internal Medicine, Inova Fairfax Medical Center, Falls Church, VA, USA; b University of Virginia School of Medicine, Charlottesville, VA, USA; c Department of Medical Critical Care Services, Inova Fairfax Medical Center, Fairfax, VA, USA; d Inova Schar Cancer Center, Inova Fairfax Medical Center, Fairfax, VA, USA

**Keywords:** CAR-T, Neurotoxicity, Cyclophosphamide, ICANS

## Abstract

Immune effector cell-associated neurotoxicity syndrome (ICANS) is a serious complication of chimeric antigen receptor T-cell (CAR-T) therapy, associated with significant morbidity and mortality. While corticosteroids and anakinra are cornerstones of treatment, a subset of patients develop severe, steroid-refractory ICANS, highlighting a critical need for more effective therapies. We present the case of a 51-year-old male with relapsed/refractory Philadelphia chromosome-positive (Ph+) B-cell acute lymphoblastic leukemia (B-ALL) who developed grade 4 ICANS following brexucabtagene autoleucel CAR-T therapy. His neurotoxicity was refractory to high-dose corticosteroids, anakinra, and intrathecal chemotherapy. Following administration of low-dose cyclophosphamide (375 mg/m^2^), patient achieved full neurological recovery. This case suggests that earlier, lower-dose cyclophosphamide may be an effective strategy to mitigate ICANS while preserving CAR-T function, warranting further investigation to define its role in treatment algorithms.

## Introduction

Chimeric antigen receptor T-cell (CAR-T) therapy has revolutionized the treatment of relapsed and/or refractory hematologic malignancies [1, 2]. However, its efficacy is counterbalanced by unique toxicities, primarily cytokine release syndrome (CRS) and immune effector cell-associated neurotoxicity syndrome (ICANS) [3, 4]. ICANS management remains challenging; despite a multidisciplinary approach and standard immunosuppression with corticosteroids and anakinra, outcomes for severe, refractory cases are poor, leading to prolonged hospitalization, permanent neurological damage, and high mortality [5]. This underscores an urgent need for more effective treatment strategies. The evidence for salvage therapies is limited to small series and case reports. We present a case of severe, steroid-refractory ICANS successfully treated with lower-dose cyclophosphamide, resulting in rapid and complete neurological recovery. We emphasize how lower-dose cyclophosphamide is not only novel but may offer a safety advantage over prior salvage regimens for patients suffering from ICANS.

## Case Report

### Investigations

A 51-year-old male with relapsed/refractory Ph+ B-ALL, previously treated with tyrosine kinase inhibitors and a pediatric-inspired regimen, was admitted for brexucabtagene autoleucel dosed at 8.8 × 10^7^/kg. His pre-infusion workup revealed minimal residual disease (MRD) positivity without central nervous system (CNS) involvement. Lymphodepletion was performed with fludarabine and cyclophosphamide per standard protocol.

On day +5, he developed grade 1 CRS (fever to 39.1 °C). This progressed to grade 2 (hypotension responsive to fluids) on day +6, managed with three doses of tocilizumab. On day +7, he developed expressive aphasia and handwriting changes (immune effector cell-associated encephalopathy (ICE) score 9/10, grade 2 ICANS), which rapidly deteriorated to agraphia and acalculia (ICE score 6/10). He was transferred to the intensive care unit (ICU) for continuous electroencephalogram (EEG) monitoring and management. Brain magnetic resonance imaging (MRI) showed no acute abnormalities.

Despite initiation of high-dose glucocorticoid and anakinra, his ICANS progressed to grade 4 by day +10, necessitating elective intubation for agitation and to facilitate a diagnostic lumbar puncture. Cerebrospinal fluid (CSF) analysis showed mild lymphocytic pleocytosis (white blood cell (WBC) 13/mm^3^), elevated protein (67 mg/dL), and normal glucose. All infectious studies (culture, polymerase chain reaction (PCR), cytology) were negative. Intrathecal methotrexate 12 mg, cytarabine 50 mg, and hydrocortisone 50 mg were administered as the third line of treatment.

### Workup and diagnosis

The leading diagnosis was severe, steroid-refractory ICANS, with alternative considerations including CNS infection. CSF studies, including Gram stain, culture, meningitis and encephalitis panels, cryptococcal antigen, and mycobacterial PCR, were all negative. Flow cytometry and cytology of the CSF revealed no evidence of malignant cells. The patient was at increased risk for ICANS due to preceding CRS and the type of CAR-T product he received. Both initial and repeat brain MRI 9 days later showed no acute abnormalities. Continuous EEG monitoring demonstrated no evidence of seizure activity.

### Treatment and outcomes

**Figure 1:**
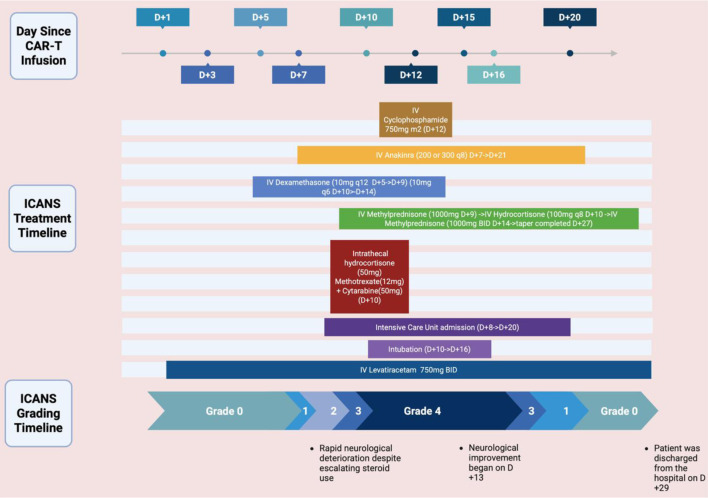
Timeline of the case including treatments administered and ICANS grading. Created in BioRender. https://BioRender.com/6uhz4jb

Due to refractory grade 4 ICANS, intravenous cyclophosphamide (375 mg/m^2^) was administered on day +12. Within 24 h, the patient’s mental status began to improve. He was successfully extubated on day +16 and achieved full neurological recovery by day +19. High-dose corticosteroids were tapered off by day +27 without rebound neurotoxicity. Figure 1 outlines the timeline of this case.

## Discussion

ICANS is a potentially life-threatening complication of CAR-T therapy. This syndrome typically manifests within the first 1 - 2 weeks post-infusion, often coinciding with or shortly following the peak of CRS [6]. A meta-analysis from 2024 encompassing 75 trials and over 3,000 patients reported a pooled ICANS incidence of approximately 27% and a severe ICANS incidence of about 10%, with considerable variation across CAR T-cell products and patient populations [7]. Anti-CD19 constructs, particularly those with CD28 costimulatory domains, consistently show higher rates of both all-grade and high-grade ICANS than anti-B-cell maturation antigen (anti-BCMA) constructs. The clinical presentation of ICANS is remarkably heterogeneous, ranging from subtle cognitive deficits, such as impaired attention, dysgraphia, and mild expressive aphasia, to severe and life-threatening complications including global aphasia, seizures, obtundation, cerebral edema, and, in rare instances, coma [8]. Motor findings such as tremor, dysmetria, or myoclonus may also occur [8]. The patient described in this report exhibited a classic progression of these symptoms, beginning with agitation and handwriting changes and rapidly advancing to grade 4 ICANS. ICANS after CAR-T therapy is driven by a combination of disease, treatment, and patient-related risk factors. Severe or early onset CRS as well as high tumor burden are two of the strongest predictors of ICANS [9, 10]. Patient-related risk factors such as age, performance status, pre-existing neurological disease, and coagulopathy also increase vulnerability [11]. Treatment with a higher CAR-T cell dose, rapid CAR-T expansion, and constructs with CD28 co-stimulatory domains, such as brexucabtagene autoleucel, all put patients at increased risk for ICANS [10]. CD28-based CAR T cells expand more rapidly and produce a more intense cytokine surge, leading to greater endothelial activation and blood-brain barrier disruption. Regarding this case, the major risk factors for this patient were his development of CRS and the type of CAR-T cell product received.

The diagnostic approach to ICANS aims to both grade the severity and rule out differential diagnoses such as infectious encephalitis, metabolic encephalopathy, or disease progression. The ICE score is a standardized tool for bedside assessment of cognitive function [12]. Further workup typically includes EEG, which may reveal non-specific findings like generalized slowing or, less commonly, seizure activity; neuroimaging with MRI, which is often unremarkable in early stages but may show signs of edema or leukoencephalopathy in severe cases; and CSF analysis, which frequently demonstrates a mild lymphocytic pleocytosis and elevated protein, serving primarily to exclude other etiologies.

The management of ICANS is stratified based on the severity of the presentation, as outlined by consensus guidelines [12]. For grade 1 ICANS, supportive care and close monitoring are often sufficient. Grade 2 or higher typically necessitates the initiation of corticosteroids, with dexamethasone being a common first-line choice. High-grade ICANS (grades 3-4) constitute a medical emergency, requiring management in an intensive care unit, escalation to high-dose intravenous corticosteroids (e.g., methylprednisolone 1 - 2 mg/kg/day), and seizure prophylaxis.

A critical nuance in management is the limited role of tocilizumab in isolated ICANS; while paramount for treating systemic CRS, its large molecular size impedes CNS penetration, and its use can paradoxically elevate circulating interleukin-6 (IL-6) levels, which may potentially exacerbate neuroinflammation [12]. For cases refractory to initial steroid therapy, treatment options are less defined and are based on limited evidence. Options with emerging but limited evidence include the IL-1 receptor antagonist anakinra, the IL-6 ligand inhibitor siltuximab, and intrathecal chemotherapy [12]. New lines of treatment targeting endothelial activation and earlier IL-1 blockade are under active investigation to reduce the need for prolonged steroid exposure [13]. The absence of a robust, evidence-based standard of care for steroid-refractory ICANS represents a significant unmet need and is associated with high morbidity and mortality.

The use of cyclophosphamide in this context is supported by its mechanism as an alkylating agent, which targets proliferating immune cells implicated in the inflammatory cascade of ICANS. Cyclophosphamide is a cost-effective and widely available agent, offering a practical alternative to more expensive or less accessible therapies such as siltuximab and ruxolitinib, particularly in urgent or resource-limited settings. Existing literature on its use is limited to case reports, where it is typically employed as a salvage therapy at high doses (e.g., 750 to 1.5 g/m^2^) with associated risks of significant cytopenias and infectious complications [14]. Lower dosing may help mitigate the cytotoxic effects of cyclophosphamide, reducing lymphocyte depletion and thereby maintaining CAR-T persistence and function. By dampening the overactive immune response and reducing cytokine-driven endothelial activation, cyclophosphamide may help restore blood-brain barrier integrity and mitigate CNS inflammation [15]. This observation is consistent with other reports indicating that CAR-T populations may persist despite cyclophosphamide exposure [14].

Our patient had a remarkable recovery once 375 mg/m^2^ of cyclophosphamide was given despite numerous days of extended steroids, anakinra, and intrathecal chemotherapy. This case contributes to the emerging evidence on management strategies for steroid-refractory ICANS. The successful outcome with lower-dose cyclophosphamide suggests that earlier intervention at a moderated dose may be a viable therapeutic option, balancing efficacy with a potentially improved safety profile.

### Conclusion

This is a rare case report detailing the successful use of a much lower-dose cyclophosphamide for refractory ICANS in a patient with Ph+ B-ALL. This case highlights that cyclophosphamide, administered earlier in the treatment algorithm, can achieve rapid neurological resolution with a manageable safety profile. It offers a promising, practical, and cost-effective salvage strategy that may help preserve CAR-T therapy. Further prospective studies are needed to validate these findings and define the optimal dosing and timing for cyclophosphamide in the management of ICANS.

### Learning points

Severe, refractory ICANS is a neurological emergency that can lead to rapid clinical deterioration and death. The current management relies on steroids that induce immunosuppression while putting the patient at risk for other side effects. Cyclophosphamide is emerging as an option for severe ICANS that is resistant to steroid therapy and this case was a success story on its implementation. More research and data are needed for better treatment options and guidelines for this dangerous complication of CAR-T therapy, but the novelty and possible safety advantages of using low-dose cyclophosphamide to treat refractory ICANS hold promise.

## Data Availability

The authors declare that the data supporting the findings of this study are available within the article.
